# Simultaneous Faraday filtering of the Mollow triplet sidebands with the Cs-D_1_ clock transition

**DOI:** 10.1038/ncomms13632

**Published:** 2016-11-25

**Authors:** Simone Luca Portalupi, Matthias Widmann, Cornelius Nawrath, Michael Jetter, Peter Michler, Jörg Wrachtrup, Ilja Gerhardt

**Affiliations:** 1 Institut für Halbleiteroptik und Funktionelle Grenzflächen, Center for Integrated Quantum Science and Technology (IQ^*ST*^) and SCoPE, University of Stuttgart, Allmandring 3, 70569 Stuttgart, Germany; 2 3rd Institute of Physics, Center for Integrated Quantum Science and Technology (IQ^*ST*^) and SCoPE, University of Stuttgart, Pfaffenwaldring 57, D-70569 Stuttgart, Germany; 3 Max Planck Institute for Solid State Research, Heisenbergstraße 1, D-70569 Stuttgart, Germany

## Abstract

Hybrid quantum systems integrating semiconductor quantum dots (QDs) and atomic vapours become important building blocks for scalable quantum networks due to the complementary strengths of individual parts. QDs provide on-demand single-photon emission with near-unity indistinguishability comprising unprecedented brightness—while atomic vapour systems provide ultra-precise frequency standards and promise long coherence times for the storage of qubits. Spectral filtering is one of the key components for the successful link between QD photons and atoms. Here we present a tailored Faraday anomalous dispersion optical filter based on the caesium-D_1_ transition for interfacing it with a resonantly pumped QD. The presented Faraday filter enables a narrow-bandwidth (Δ*ω*=2*π* × 1 GHz) simultaneous filtering of both Mollow triplet sidebands. This result opens the way to use QDs as sources of single as well as cascaded photons in photonic quantum networks aligned to the primary frequency standard of the caesium clock transition.

## Abstract (web summary)

Hybrid quantum systems combine efficient high-quality quantum dot sources with atomic vapours that can serve as precise frequency standards or quantum memories. Here, Portalupi *et al*. demonstrate an optimized atomic Cs-Faraday filter working with single photons emitted from a semiconductor quantum dot.

## Graphical abstract

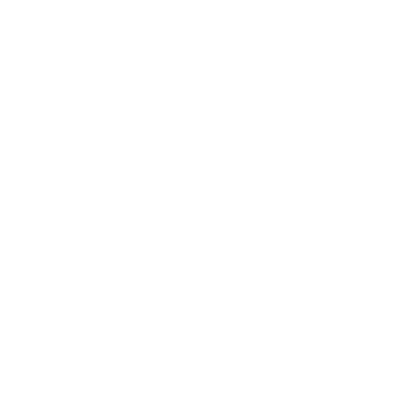

In recent years, hybrid quantum systems have received a lot of attention with the vision and perspectives of merging the strengths of complementary worlds. Initial experiments have been conducted interfacing single-photon emission from solid state sources and atoms. The latter are known for their long coherence time and nonlinear properties. For this scope, several different single-photon sources have been used, from spontaneous parametric down-conversion^1^,^2^ to single organic molecules^3^. Atomic vapours allow for the implementation of gigahertz wide filters with near-unity transmission, which are very robust against environmental influences. Several advantages come with using quantum dot (QD)-based light sources, such as the deterministic nature of the single-photon emission^4^. In addition to that, these sources demonstrated a state-of-the-art indistinguishability together with a brightness up to 20 times higher than other probabilistic sources with comparable indistinguishability^5^,^6^,^7^. All these properties render them as reliable sources for quantum optics and quantum information applications. The first demonstration of a direct photonic coupling between a quantum dot and a single trapped ion has been recently demonstrated^8^. Earlier experiments have been shown with atomic rubidium or caesium vapours interfaced with single and entangled photons generated by semiconductor quantum dots. They focused on absorptive^9^,^10^,^11^,^12^ properties and slow light^13^,^14^,^15^.

In the present paper, we take a step forward towards a universal QD–atom interface. We first determine the optimal working conditions of a caesium-based Faraday anomalous dispersion optical filter (FADOF). Then, using strong resonant pumping of the QD, we generate the so called Mollow triplet spectrum. The two arising sidebands have demonstrated the capability to emit photons in a cascaded fashion and are tunable to the caesium clock transition. We then interface them to the FADOF and simultaneously transmit the two sidebands of the Mollow triplet across it, while off-resonant light is strongly suppressed. The present results demonstrate the possibility of using single and cascaded photons from a resonantly excited QD with a detuning precisely set by the atomic transition, a fundamental precondition for the realization of quantum networks.

## Results

### The Mollow triplet

A (In,Ga)As/GaAs QD is resonantly driven with a continuous wave (CW) laser. When a two-level system is under strong resonant excitation, the simple two-level approach is no longer valid. As established in the literature^16^, we describe the system in a dressed state approach. The atom-like energy levels of the QD are then replaced by multiplets and the recombination between these dressed states gives rise to the so-called Mollow triplet^17^. The resulting spectrum, arising from strong resonant pumping, is composed of three lines: a central line, called Rayleigh (R), and two symmetric sidebands called respectively fluorescence (F) and three-photon (T) lines^18^. The photons emitted in the F and T sidebands have been demonstrated to be highly indistinguishable^19^, and can also be emitted in a cascaded manner if the QD is specifically pumped^20^. The splitting of the QD sidebands can be spectrally aligned to the atomic transitions of the Cs hyper fine splitting (that is 9.2 GHz) and their spectral position can be controlled precisely by adjusting the laser power or the QD-to-laser detuning. Increasing the laser power results in a progressive increase of the detuning of the F and T lines with respect to the central R line since the effective Rabi frequency, which determines the splitting, is changed (Supplementary Fig. 1). On the other hand, a small variation of the laser wavelength results in an overall shift of the full Mollow triplet. These two tuning parameters become very important to perform the current experiment since, as will be explained in the following, a precise and accurate tuning is required to match the narrow-band atomic filter.

### FADOF characterization and modelling

The use of alkali atoms to form narrow-band optical filters is nowadays an established technique. Faraday anomalous dispersion optical filters (FADOFs) utilize the Faraday effect in order to filter near-atom-resonant light^21^,^22^. An atomic vapour cell with an external magnetic field rotates the polarization of the input light to a crossed polarization output port. Simultaneously, the absorption of the atomic filter has to be low. An ideal point is given close to the atomic resonances when the absorption is low, but the dispersion still allows for a polarization rotation of *π*/2. Recently, these filters have demonstrated astonishing properties, such as narrow linewidths and reduced background^21^,^23^,^24^, and enabled treating the problem as an optimization challenge^25^. An interesting feature used in the present experiment is that this filter shows the option to work as a double narrow-band pass filter with the two bands separated by the Cs-clock transition at around 10 GHz, as discussed above.

**Figure 1:**
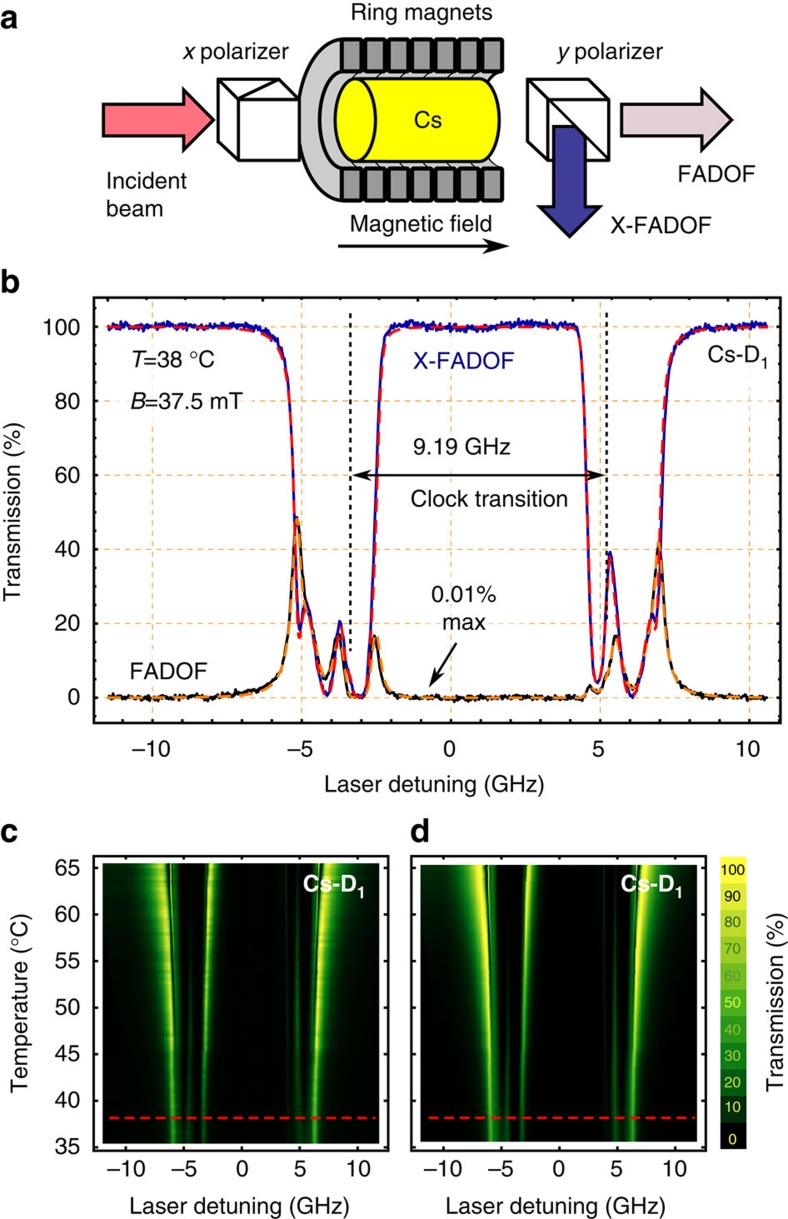
Experimental and theoretical characterization of the Faraday filter. (**a**) Experimental setup. The caesium vapour cell is placed between two crossed polarizers. A magnetic field is applied via ring shaped permanent magnets. The incident laser is polarized by the first *x* polarizer and then analysed in cross-polarized configuration after passing through the FADOF. (**b**) Transmission spectra of the Cs-D_1_ line plotted as function of the laser detuning for the FADOF and X-FADOF. Laser detuning refers to the angular frequency against the transition frequency of atomic caesium (2*π* × 335.116048807(41) THz, centre wavelength *λ*_vac_=894.593 nm). Transmission (FADOF, calculated: orange, dashed; measured: black) and the second output port (X-FADOF, calculated: red, dashed; measured: blue). When both spectra are added, the Doppler spectrum is obtained. (**c**) Measured transmission spectrum as a function of temperature and laser detuning, red dashed line indicates data shown in (**b**). (**d**) Fit of the measured transmission data shown in (**c**). All fits and calculations are performed according to ref. 27.

In this work we aim for filtering the quantum dot photons with a Faraday filter. To find the optimal working conditions, a full theoretical analysis was performed by calculating the electric susceptibilities and the subsequent filter function. In addition, the Cs-based Faraday filter was experimentally implemented and characterized with a few microwatts of a titanium-sapphire laser^26^. The experimental configuration of the FADOF is depicted in Fig. 1a. The transmission over the laser detuning is measured while changing the vapour temperature (by means of a feedback loop controlled heater as explained in methods), while keeping the magnetic field constant (at 37.5 mT). Fig. 1b–d shows the acquired FADOF spectra along with their theoretical prediction. Both output ports of the polarizing beamsplitter are monitored: if summed they represent the Doppler spectrum. The crossed output is the FADOF signal and other port is called X-FADOF (cross FADOF). To compensate for the losses of the cell windows, we assumed a far-off resonant point to transmit 100% of the light. Each spectrum was independently fitted with the program introduced in ref. 27. First, both temperature and magnetic field were set as free fit parameters. The same magnetic field of 37.5±0.5 mT was found for each temperature. This value is in good agreement with the value obtained by a Hall sensor, and was set to a fixed value for all further fits. The temperature axis plotted in Fig. 1c,d, was obtained as a free fit parameter (Supplementary Note 1). The fit for both polarization axes provided the same temperature. However, a small deviation (0.4 °C) between the actual experimental set point of the controller and the fit result was found. A common way to describe the performance of Faraday filters is the equivalent noise bandwidth, which relates the maximum transmission of the filter to the background in the spectral proximity. For the filter parameters used it is 2.97 GHz. Please note that this parameter may be misleading in this context since the filter is used as a multi-line band-pass filter.

To evaluate the electric susceptibilities of the Cs vapour^26^, the Hamiltonian *H*=*H*_0_+*H*_HFS_+*H*_Zeeman_ is calculated and results in the complex susceptibilities 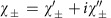. Altogether with the length of the cell, *L*, and the temperature, a Voigt line profile is obtained for each transition. The combination of optical rotation by *π*/2 and simultaneous weak Doppler absorption leads to a preferred optical configuration. The transmission 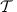 is calculated as


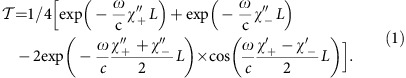


We assume the Doppler broadening and the atomic density based on the same single temperature. A more detailed study of the theoretically estimated spectra is given in, for example, ref. 28. For the theoretical model a self-developed program^29^ and additionally the package ElecSus^27^ was used. For fitting the data ElecSus was preferred for providing a fast Python-based fitting routine. The frequency axis was linearized by fitting a simultaneously recorded transmission spectrum through a Fabry-Pérot etalon with an Airy-function.

### Optimal filter configuration

**Figure 2:**
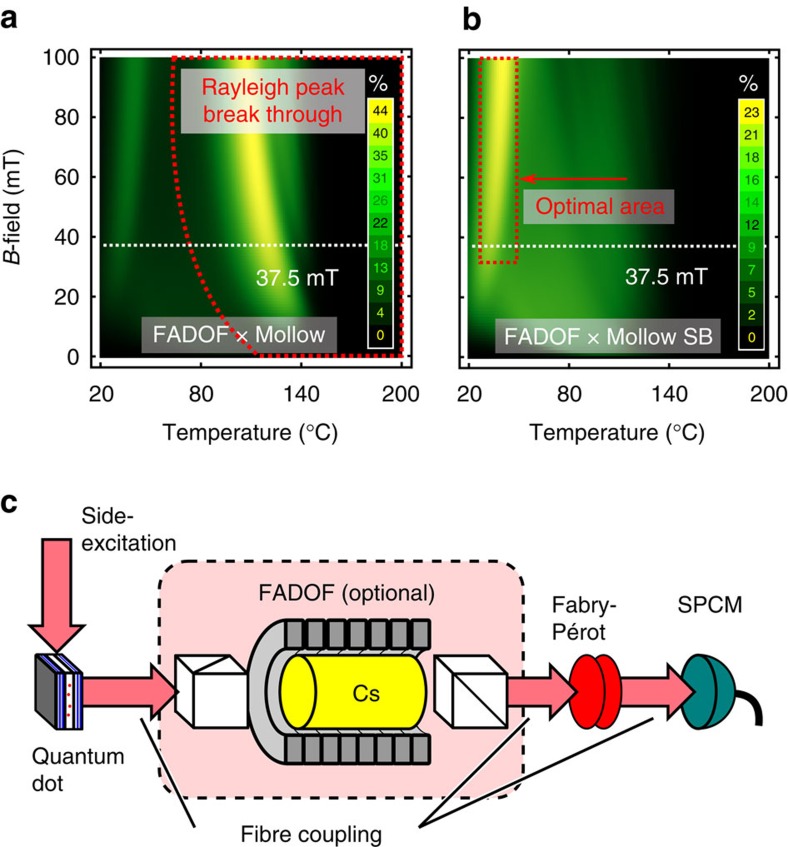
FADOF optimization and setup for the QD-atom interface description (**a**) Transmission efficiency, *η*_eff_(*B*,*T*), of the entire Mollow emission filtered by a Cs-Faraday filter. The centre (Rayleigh) peak passes through the filter if the temperature is too high (area to the right of the red dashed line). Experimental configuration, with 37.5 mT, is marked (white dashed line). (**b**) Transmission efficiency of the Mollow sidebands, optimal area for best transmission (red highlighted area). It is worth noting that the map in (**a**) is normalized over the total Mollow spectrum (that is Rayleigh line plus the two sidebands), while (**b**) is normalized over the sidebands only (that is over half of the previously used area). This means that the numbers displayed in (**b**) represent the actual value of sidebands transmission. (**c**) Sketch of the complete setup: the light from the resonantly excited QD is sent through the Faraday filter. Because all components are fibre coupled, the FADOF can be easily removed to send the QD light directly into the Fabry-Pérot inferferometer.

In the following the hybrid experiment, that is the interface between the F and T lines coming from the QD and the alkali vapour FADOF, will be reported and described. The actual experiment has been simulated with the same theoretical means described before. By the given signal *S* from a single QD, the transmission of the sidebands through the filter needs to be optimized whereas the resonant scattering (middle peak) needs to be suppressed. To analyse it, a multiplication of the transmission function 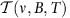 and Mollow triplet sidebands *S*^Mollow SB^ is required:


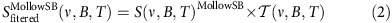


where 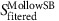 describes the filtered Mollow sideband signal, as a function of the detection frequency *ν*, the magnetic field *B* and the temperature *T*. This can be used to define an integrated transmission efficiency: 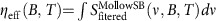. Note that the unit is the percentage of total transmission, and due to the limitation to the sidebands, this represents a number between 0 and 50%, as shown in Fig. 2a. On the other hand, Fig. 2b shows the values when only the Mollow triplet sidebands are taken into account. The normalization is then performed over an area equal to the half of the previous one (that is limited to the sidebands), resulting in the expected filter transmission. By varying the temperature (20–200 °C) and the B-field (0–50 mT), we have found an optimal working area. Specifically, we found the best *η*_eff_ between 25 and 65 °C, and above 25 mT, as illustrated in Fig. 2b.

### QD photons interfaced with alkali atoms

In the experiment, the FADOF was operated at a temperature of 28 °C since these are the proper working conditions, as it can be seen by comparing Fig. 2a,b. To suppress the middle Rayleigh peak the filter has to be kept below 40 °C. *B*-fields do not dramatically increase the efficiency, *η*_eff_, above 25 mT. By the given magnetic field of 37.5 mT, set by the utilized permanent magnets (see Methods), the optimal temperature for a maximized transmission of the sidebands is found to be 34 °C. Despite that, the temperature in the experiment was set to 28 °C in order to better suppress the central Rayleigh peak without dramatically affecting the transmission (Supplementary Fig. 2). The QD sample consists of a layer of single (In,Ga)As/GaAs quantum dots that are placed inside a planar cavity structure (see Methods). The sample is then kept at 4 K using a standard continuous-flow helium cryostat. The QD used in the experiment is selected in order to have the *s*-shell transition around the intended wavelength (*λ*_vac_=894.593 nm) and then excited resonantly. The continuous wave resonant laser is sent onto the sample side while the QD emission is collected from the top of the sample^30^,^31^. This configuration helps reducing the amount of resonant laser collected and this is also further suppressed by means of a crossed polarized detection. The QD signal is then coupled into a polarization maintaining single-mode (SM) fibre. This light can then be sent into a scanning Fabry–Pérot interferometer (FPI) with a resolution of 0.1 GHz coupled with a conventional single-photon counting module (SPCM)^32^: this setup allows resolving the Mollow triplet spectra before and after the interface with Cs atoms. Since the FADOF also has fibre coupled input and output, it can be easily inserted between the source (that is the QD) and the detection (that is the FPI). A sketch of the current setup is shown in Fig. 2c. Thanks to the aforementioned tuning parameters of the emitted Mollow triplet (that is laser power and wavelength), the sidebands can be carefully tuned to match the Cs-D_1_ transitions.

**Figure 3:**
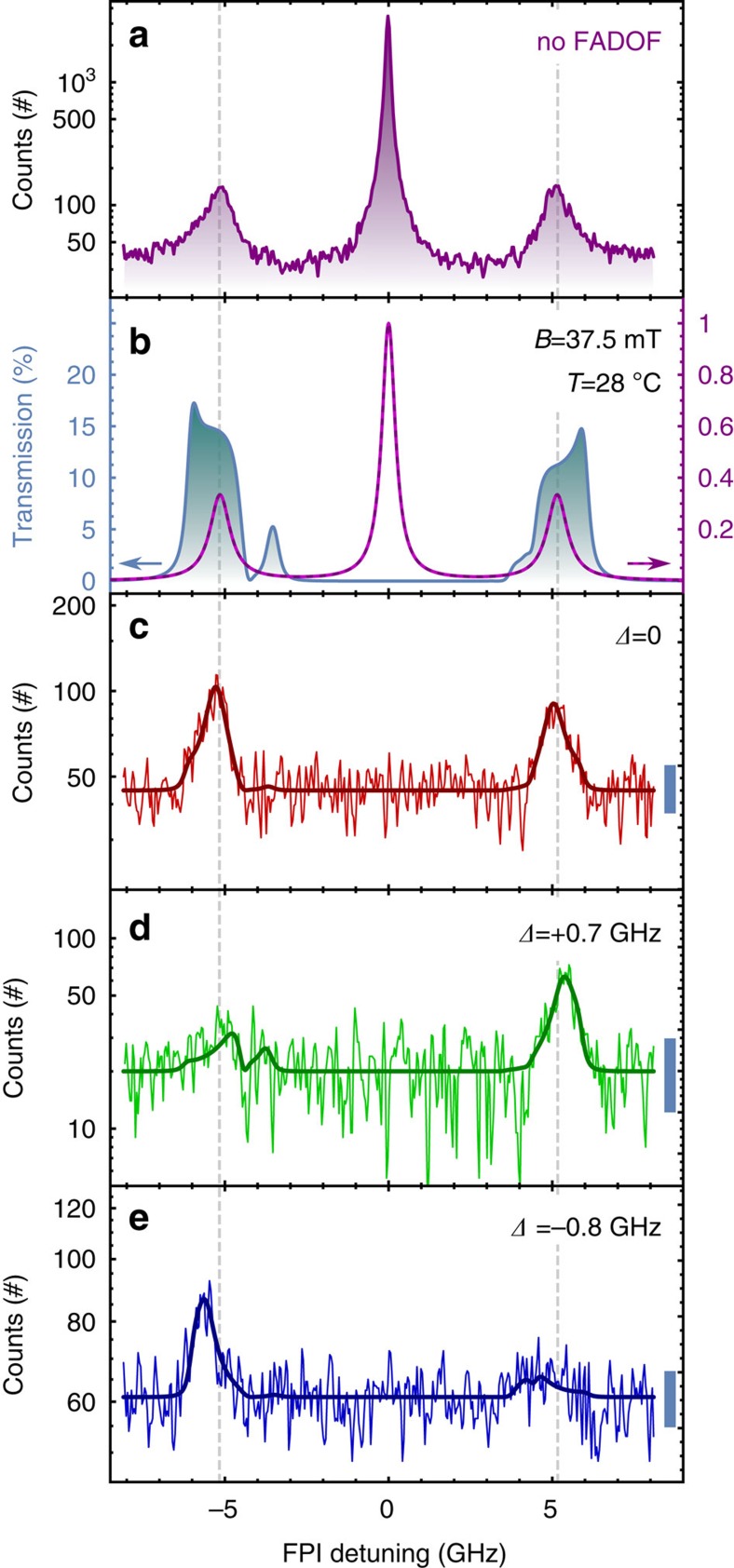
Mollow triplet sidebands filtered by the FADOF (**a**) Mollow triplet with both sidebands tuned on resonance with the filter transmission features (dotted lines): integration time around 300 s. (**b**) Calculated Mollow triplet (purple) and calculated filter function of the Cs-FADOF (blue): (**c**) Mollow triplet spectrum transmitted through the FADOF: both sidebands are simultaneously transmitted while the central Rayleigh peak is fully suppressed. Integration time around 4000, s. The solid curve is calculated as a simple multiplication of the spectra in (**b**), normalized considering the current count rate. (**d**) Experiment performed after detuning the laser (so also the Mollow triplet) by +0.7 GHz and with 90% of the previous pump power: only one sideband is now transmitted through the filter. Solid green curve represents the calculation with the actual experimental parameters (that is power and detuning) (**e**) Same experiment as in (**c**) but with a laser detuned by −0.8 GHz and with same power as in (**d**). For both (**d**) and (**e**) the integration time was around 2000, s. The shot noise is displayed by the blue bars on the right side of panels (**c**)–(**e**). More information can be found in Supplementary Information.

The Mollow triplet obtained by setting the laser at *λ*_vac_=894.593 nm is displayed in purple in Fig. 3a: it is worth noting that the F and T sidebands are now matched with the known transmission features of the FADOF (central wavelength is highlighted by grey dashed lines). Fig. 3b shows the calculated transmission spectrum of the FADOF and the Mollow triplet emission in the present configuration. A simple multiplication of the two spectra gives a representation of the current experiment. The deviation between the measured and calculated Mollow triplet is the coherent scattering fraction, which forms a narrow-line at zero detuning. Measuring the spectrum after the filter results in the red curve in Fig. 3c. Only the F and T lines are transmitted, being resonant with the Cs-D_1_ transition. On the other hand the central peak, consisting of the Rayleigh line and residual laser scattering, is shown to be suppressed to background level by more than a factor of 100. This is equivalently shown in the laser-based experiments, depicted in Fig. 1. The calculated suppression exceeds 40 dB over a range of more than 4.1 GHz, essentially centred between the two ground states of caesium. If instead of using the FPI, the atomic-filtered QD light is sent directly to the SPCM, we obtain for both sidebands 2,000 cps (counts per second). Considering the observed intensity on the two sidebands in comparison with previous studies^20^, we now assume a reasonable value of 10^5^ detectable photons per second emitted by the QD. These photons are distributed over the three transitions (1/2 on the Rayleigh line, 1/4 per sideband). Subsequently, the overall transmission is expected to be between 10 and 15% of the integrated sidebands with 6,000 cps passing the filter in total; this corresponds to the integrated area under the transmission curve in Fig. 3c. The slight discrepancy with the measured value can be due to experimental losses or a slightly less bright QD. Comparing the areas of the sidebands in Fig. 3a,c, normalized on the integration time, the measured transmission appears to be around 10% (see Supplementary Note 2 for more details on the integration time). Novel developments in Faraday filtering, such as the application of a non-collinear magnetic field, as described in ref. 22, pave the way to more efficient filters in the future. This experiment represents, to the best of our knowledge, the first demonstration of the simultaneous filtering by an atomic vapour of two resonance fluorescence lines from a single QD with a different frequency (F and T) and with a detuning defined by the atomic clock transition. The solid line in Fig. 3c represents the overall transmitted spectrum and agrees very well with experimental data. Even the small features giving rise to a slight asymmetry in the transmitted spectrum are clearly visible.

In order to demonstrate that such a resonant condition for the hybrid interface is stringent, we performed the same experiment changing the laser detuning and with a laser power reduced to 90%. Varying the laser frequency by +0.7 GHz, only a fraction of the T line is now resonant with the Cs-transitions. This results in the spectrum displayed in Fig. 3d where only one peak is observed. On the other hand, a frequency decrease of −0.8 GHz shows that only a fraction of the F line is then transmitted giving the spectrum in Fig. 3e. For all experiments, the shot noise is displayed showing that we are well above such noise level. With the same calculations, using the current settings of laser wavelengths and power result in the solid green and blue lines in Fig. 3d,e, respectively. The transmission peak that is not in clear resonance with the Mollow sideband is expected to have a much lower intensity and, even if it is experimentally visible, it is only slightly higher than the shot noise. These experiments demonstrate another strength of the hybrid QD-alkali system: the light emission has to be carefully tuned to match the atomic transitions and these clock-frequencies are universal. This represents a very interesting perspective for the implementation of quantum repeaters and quantum networks, since the wavelength of the photons is set by the atomic properties, being then an easy to handle and absolute reference point.

## Discussion

In conclusion, we successfully demonstrated an interface between photons coming from the two sidebands of a resonantly-driven QD and an alkali-atom vapour. The introduced theoretical model allowed us to estimate the optimal operation conditions of the Faraday filter and fully describes the conducted hybrid experiment. The FADOF works as a double-band narrow filter simultaneously for both photons (from the F and T lines), posing a strict condition on the wavelength detuning that can be used in the development of quantum networks as precise and universal reference set by the atomic clock transitions. The gigahertz-wide filter introduced here does not suffer from environmental influences such as mechanical drift and is automatically aligned to an atomic transition. The presented results show the perspectives for the implementation of more complex experiments benefiting from the strengths of these hybrid systems like locking the QD emission to an atomic clock transition or storing a photon from the quantum dot into the alkali vapour.

## Methods

### FADOF characterization

A few microwatts of a narrow-band titanium-sapphire laser (TiSa, 899-21, Coherent, CA) light were delivered by an optical single-mode fibre to the experiment. The beam waist was 2*w*_0_=4.5 mm. The frequency detuning was monitored by a Fabry–Pérot cavity and the incident laser power into the experiment was monitored by an amplified photodiode. The polarization state of the light was then fixed with a Glan-Taylor polarizer (Thorlabs GT10-B) and passed through an evacuated anti-reflection coated caesium vapour cell (coated inside and outside, Triad Technologies, CO), with an optical path length of 100 mm. The cell was externally heated with copper blocks at the optical windows by a feedback loop controlled heater. The temperature stability was estimated to be better than 0.5 °C. The FADOF transmission was analysed by photodiodes behind a Glan-Taylor calcite prism. Both output ports were monitored. If summed they represent the Doppler spectrum. For convenient data acquisition, all signals were recorded by an oscilloscope triggered by the laser control box. A full data set was acquired with temperatures ranging from 35 to 70 °C. Eight axial magnetized ring-magnets (outer diameter × bore diameter × thickness=100 × 60 × 20 mm^3^) introduced a homogeneous longitudinal magnetic field of 37.5 mT. The homogeneity was calculated to be better than 2% in the inner 80 mm and deviated maximally 5% on the outer 10 mm (Supplementary Fig. 3 for more details). The field was estimated by a commercial Gauss metre and the atomic spectra, which were acquired earlier with a solenoid configuration. The field inhomogeneity was estimated by a calculation of the experimental configuration with a numerical solver (Comsol Multiphysics 4.4). The ferrite based magnets retain their magnetization up to 250 °C, and were thus suitable also for higher temperature experiments. Comparable configurations with permanent magnets were reported earlier, such as for example in a high-field Hallbach configuration^33^.

### Sample description

The QD sample consists of a single layer of (In,Ga)As/GaAs quantum dots grown by metal-organic vapour-phase epitaxy. Two distributed Bragg reflector (DBR) layers surround the QDs that are placed in the middle of a GaAs *λ*-cavity. The inhomogeneously broadened QD distribution ranges from 885 to 910 nm. Further information regarding the sample or the experimental conditions can be found in ref. 20.

### Data Availability

All data is available on request to corresponding authors.

## Additional information

**How to cite this article**: Portalupi, S. L. *et al*. Simultaneous Faraday filtering of the Mollow triplet sidebands with the Cs-D1 clock transition. *Nat. Commun.* **7**, 13632 doi: 10.1038/ncomms13632 (2016).

**Publisher's note**: Springer Nature remains neutral with regard to jurisdictional claims in published maps and institutional affiliations.

## Supplementary Material

Supplementary Information Supplementary Figures 1-3, Supplementary Notes 1-2 and Supplementary References
