# Supplementary material for: Simultaneous Faraday filtering of the Mollow triplet sidebands with the Cs-D1 clock transition
Source: Nat Commun. 2016 Nov 25;7:13632. doi: 10.1038/ncomms13632 (PMC5133695; doi:10.1038/ncomms13632)
Supplement: Supplementary Information — Supplementary Figures 1-3, Supplementary Notes 1-2 and Supplementary References [file ncomms13632-s1.pdf]

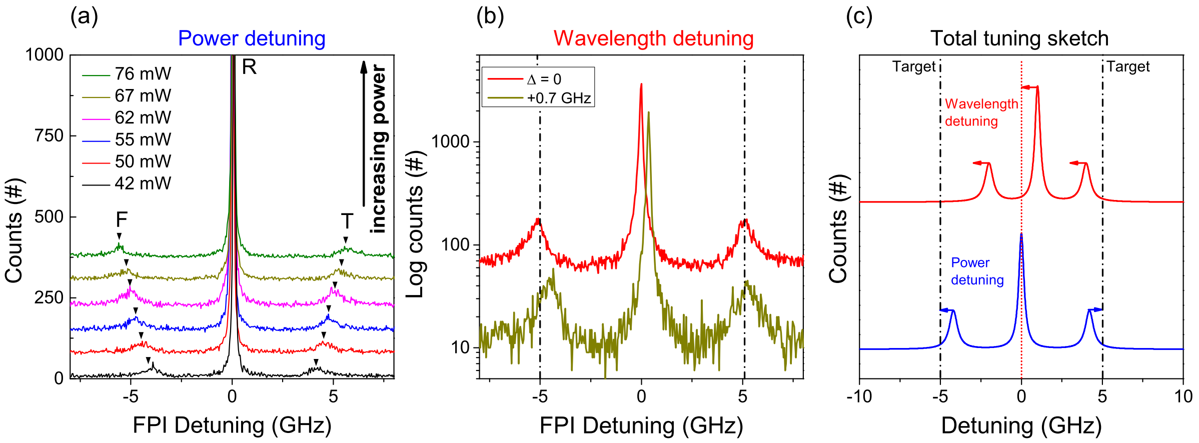

Supplementary Figure 1 | Mollow triplet tuning mechanisms (a) Spectral dependence of the Mollow triplet as a function of the laser power. (b) Mollow triplet spectra for two different values of the laser detuning, i.e. $\Delta = 0$ (red) and $\Delta = +0.7$ GHz (yellow) (c) Sketch of the two tuning mechanisms to match the desired wavelengths. Dash-dot lines in (b) and (c) refer to the target frequency corresponding to cesium transitions. As shown in the main text, the present experiment requires an exact matching of the emitted photons wavelength with the atomic resonances. To achieve that, the Mollow triplet has two different tuning mechanism that are used. Once the laser wavelength is set, an increase of power leads to a simultaneous shift of the F and T lines as shown in (a). An accurate control of the laser power allows very precise tuning of the sidebands with respect to the central R line. In addition, as investigated in Supplementary Reference [1], also the laser wavelength plays a role in the spectral position of the Mollow triplet, resulting in a shift of all three R, F and T lines. As a matter of example, two Mollow triplets are shown in (b). A variation of around -2 pm in the laser wavelength results in an overall shift of the triplet spectrum. With these two tuning knobs, it is then possible to finely adjust the sidebands spectral position in order to match the desired wavelengths, in the present case the Cs-D_1 clock transition, as sketched in (c).

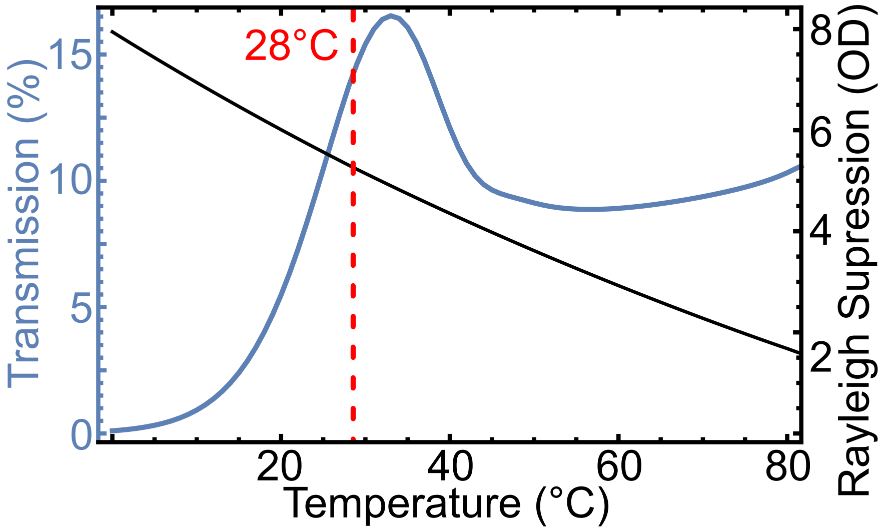

Supplementary Figure 2 | FADOF transmission and Rayleigh line suppression versus temperature Calculated filter transmission for the entire Mollow triplet (blue curve, left scale) and Rayleigh rejection (black curve, right scale) as a function of temperature. The transmission curve represents the dotted white line in Figure 2a and sets as 100% the total transmission of both sidebands. The suppression curve is the Mollow triplet integrated on a centered 4.0 GHz window and multiplied with the Faraday filter. The experimental working point is displayed as a dashed red line. The maximal transmission is at 34°C. Despite that, in the experiment we have chosen to work at a slightly lower temperature (28°C) in order to increase the Rayleigh line rejection without strongly affecting the sidebands transmission. The signal increase for temperatures above 60°C is due to the Rayleigh component breakthrough.

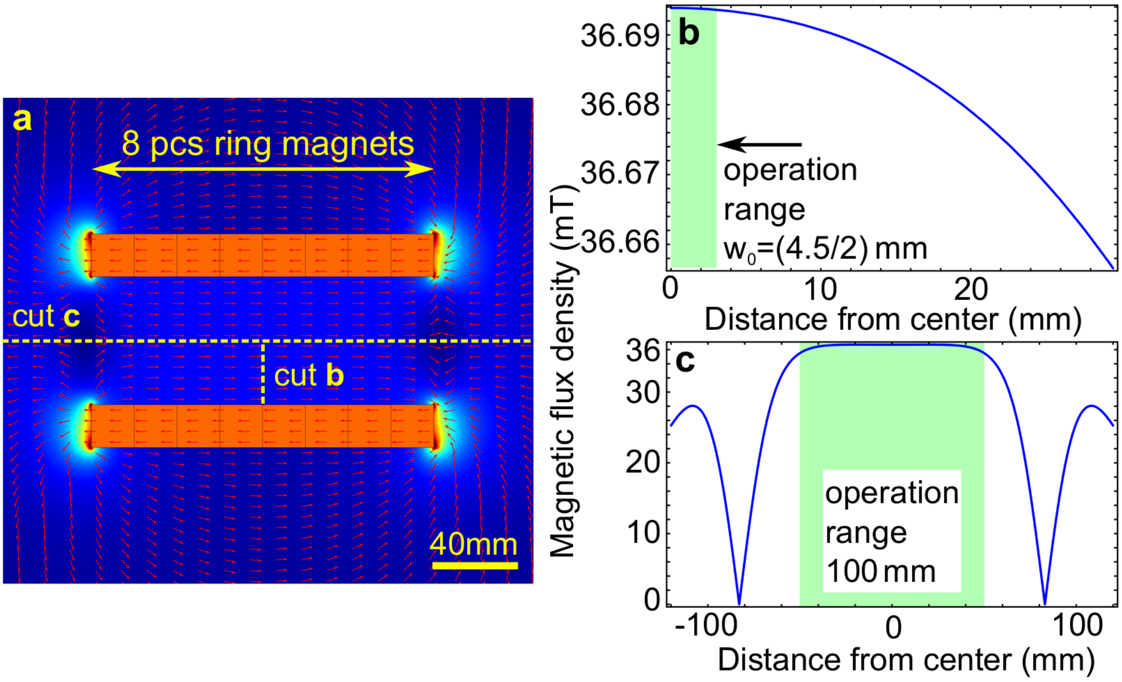

Supplementary Figure 3 | Homogeneity of the magnetic field (a) Numerical modelling of the ring magnets around the Cs cell. (b) magnetic field radial from the centre. We ensured that the beam was passing very precisely along the axes of the Cs cell. (c) Axial cut along the cesium cell. Here inhomogeneities are evident. The (simulated) field of around 36 mT is reduced in the outer 10 mm by approx. 3%. Please note, that the exact magnetic flux density of the material was unknown due to missing specifications. This plot therefore only allows to estimate the inhomogeneity and not the absolute magnitude of the magnetic flux density. The homogeneity is calculated to be better than 2% in the inner 80 mm and deviates maximally 5% on the outer 10 mm.

Supplementary Note 1

Vapour temperature determination The "perfect" thermalization of vapour cells is commonly an issue. We had two ways of determining the temperature: One was by setting it at the external temperature controller and naively reading off the value, the second was to fit the acquired spectrum. The temperature controller we used controls the temperature of two copper blocks, which are heating the cell at the optical windows. For simplicity this was omitted in Figure 1. In between, the cell is in an air compartment and so slightly cooler. Convection was suppressed by an outer housing also holding the magnets in place. The system was in a steady state after a certain thermalization time (on a tens of minutes timescale), proved by solely statistical measured temperature fluctuations around the set-point. We then compared the fit result of the fitting program (ElecSus, Supplementary Reference [2]) and the set-point. We did not perform a statistical analysis there, and no directly evident trend was observed, when we plotted the deviation. The mean of the difference was 0.4°C. We were positively surprised by the very small deviation, and believe this was caused due to the proximity to room temperature.

Supplementary Note 2

Integration time for Figure 3a, c, d and e in main text The spectra were integrated for around 300 s (a), for around 4000 s (c), and for around 2000 s (d and e). We tried to stick with the raw data as much as possible (integrated clicks on the single photon detector). Subsequently after all losses and after the FPI we obtained a number of counts per second compatible with Supplementary Ref. [1].

Supplementary References

[1] Ulhaq, *et al.* Cascaded single-photon emission from the Mollow triplet sidebands of a quantum dot. *Nature Photonics* **6**, 238-242 (2012).

[2] Zentile, M. A. et al. ElecSus: A program to calculate the electric susceptibility of an atomic

ensemble. *Comput. Phys. Commun.* **189**, 162-174 (2015).
